# Effects of azithromycin in severe eosinophilic asthma with concomitant monoclonal antibody treatment

**DOI:** 10.1136/thorax-2024-221977

**Published:** 2024-12-18

**Authors:** Gabriel Lavoie, Imran Howell, James Melhorn, Catherine Borg, Laura Bermejo-Sanchez, Jack Seymour, Maisha F. Jabeen, Anastasia Fries, Gareth Hynes, Ian D Pavord, Nayia Petousi, Timothy SC Hinks

**Affiliations:** 1 Respiratory Medicine Unit and NIHR Oxford Biomedical Research Centre, Nuffield Department of Clinical Medicine, University of Oxford, Oxford, UK

**Keywords:** Asthma, Asthma Pharmacology, Bacterial Infection, Respiratory Infection

## Abstract

Macrolides reduce exacerbations when added to inhaled therapy in severe asthma. However, there is little published evidence for effectiveness in patients treated with biologics. We conducted a retrospective audit of all patients who started azithromycin while on biologics in our centre. Compared with those that did not start azithromycin, these individuals had more exacerbations and a phenotype of chronic bronchitis and/or frequent purulent exacerbations. The addition of azithromycin to biologics was associated with reduced annual rates of steroid-treated and antibiotic-treated exacerbations and improved symptom scores (Asthma Control Questionnaire-5) but not with any improvement in lung function. Data support testing azithromycin in clinical trials in patients on biologics with residual exacerbations.

## Introduction

 Monoclonal antibodies (biologics) have revolutionised the management of severe type 2 asthma. However, some patients still experience exacerbations on treatment. Around half of these are not associated with type 2 inflammation, and most of these have viral or bacterial triggers.^1^ Azithromycin reduces exacerbations in patients with severe asthma with both low and high type 2 inflammation^2 3^ and is recommended as an add-on therapy to optimal treatment.^4 5^ In our clinical practice, we consider azithromycin as an add-on treatment in patients on biologics and otherwise optimised asthma therapy who have residual exacerbations despite a good clinical response to biologics, although evidence of efficacy in this population is lacking. In this study, we assess whether this provides added benefit in this patient group.

## Methods

### Study population

We performed an audit of all individuals with asthma treated with biologics under the Oxford Special Airways Clinic between 2008 and 2023 to identify a cohort of patients who started long-term azithromycin therapy (≥3 months) after the start of biologics. To allow comparison of baseline characteristic between this group and the rest of our patients with severe asthma treated with biologics, a comparator group was built by randomly selecting 150 individuals from the rest of the patient population. After excluding any that had macrolides prior to biologics or had missing follow-up data in the first year of biologic therapy, individuals were ordered chronologically from date of starting biologics and stratified into blocks of 5. A random number generator was used to select a subject within each of these blocks.

### Data collection

Data were obtained from electronic patient records. Rates of treatment with oral corticosteroids (OCS) and/or antibiotics were collected during routine asthma follow-up and used as surrogates for exacerbations (events). Multiple courses of treatment for the same episode were counted once. Annualised event rates for the period between the start of biologics and the start of azithromycin and between the start of azithromycin and the last review available were calculated separately for OCS-treated and antibiotic-treated exacerbations. If the interval between the start of biologics and the start of azithromycin was under 12 months, the unadjusted number of events was used to limit excessive regression to the mean. Co-primary outcomes were differences in rates of OCS-requiring events and antibiotics-requiring events after azithromycin. Secondary outcomes were differences in Asthma Control Questionnaire-5 (ACQ-5) and forced expiratory volume in one second (FEV_1_).

### Statistical analysis

Differences between groups were evaluated using a Student’s t-test or a Mann-Whitney U test for normally and non-normally distributed data, respectively. Wilcoxon signed rank tests were used to assess the change in exacerbations, lung function and ACQ-5 following treatment. The statistical significance level was set at 0.05.

## Results

Of 818 individuals on biologics, 50 started azithromycin after biologic treatment. Baseline characteristics are presented in table 1. Individuals who started azithromycin were older (mean 61.7 vs 55.5 years; mean difference 5.7; 95% CI 0.8 to 10.6) and had more OCS-treated exacerbations (median 6 vs 4 events/year; difference between medians 1.0; 95% CI 0.0 to 2.0). The prevalence of chronic bronchitis and/or recurrent purulent exacerbations was also higher (94% vs 21%, difference of proportions 73%; 95% CI 63.4% to 82.0%).

**Table 1:** Baseline patient characteristics

Characteristic	Azithromycin started (n=50)	Control group (n=150)
Age (years)mean±SD	61.7±12.9*	55.5±15.6*
Female sexNo. of patients (%)	30 (60%)	85 (57%)
Smoking statusNo. of patients (%)		
Never smoker	25 (50%)	103 (69%)
Ex-smoker	18 (36%)	31 (21%)
Active smoker	1 (2%)	0 (0%)
Not reported	6 (12%)	16 (11%)
Smoking history (pack years)mean±SD	17±15	16±14
Clinical phenotypeNo. of patients (%)		
Chronic bronchitis	32 (64%)**	24 (16%)**
Recurrent purulent exacerbations	41 (82%)**	13 (8.7%)**
Bronchitis or purulent exacerbations	47 (94%)**	32 (21%)**
Bronchiectasis on CT scanNo. of patients (%)		
Bronchiectasis	10 (20%)	16 (11%)
No bronchiectasis	40 (80%)	134 (89%)
Sputum culture		
*Haemophilus influenzae*	24 (48%)	
*Pseudomonas aeruginosa*	6 (12%)	
*Moraxella catarrhalis*	2 (4%)	
*Streptococcus pneumoniae*	1 (2%)	
*Staphylococcus aureus*	1 (2%)	
*Streptococcus viridans*	1 (2%)	
Oropharyngeal flora	8 (16%)	
No culture available	7 (14%)	
Biologic when macrolides startedNo. of patients (%)		
Mepolizumab	29 (58%)	84 (63%)
Benralizumab	19 (38%)	39 (29%)
Dupilumab	2 (4%)	0 (0%)
Omalizumab	0 (0%)	10 (7.5%)
Maintenance systemic steroids at start of biologicsNo. of patients (%)	18 (36%)	32 (38%)
Dose of OCS if on maintenance (mg) mean±SD	8.1±2.4	10.6±4.8
Blood eosinophil count (x10^9^ cells/L*median (IQR)	0.55 (0.42 to 0.79)	0.68 (0.5 to 1.01)
Exhaled NO (FeNO)median (IQR)	27.5 (19.0 to 57.5)	38.5 (21.0 to 67.5)
Steroid-requiring exacerbations in 12 months prebiologics (median, IQR)	6 (4 to 7)*	4 (3 to 6)*
Antibiotic-requiring exacerbations prebiologics (median, IQR)	3 (1 to 5)	3 (0 to 4)

*p<0.05 **p<0.001.

* rRefers to the maximum blood eosinophil count in the 12 months prior to commencing biologics; *p **p.

FeNOfractional exhaled nitric oxideOCSoral corticosteroids

**Figure 1:**
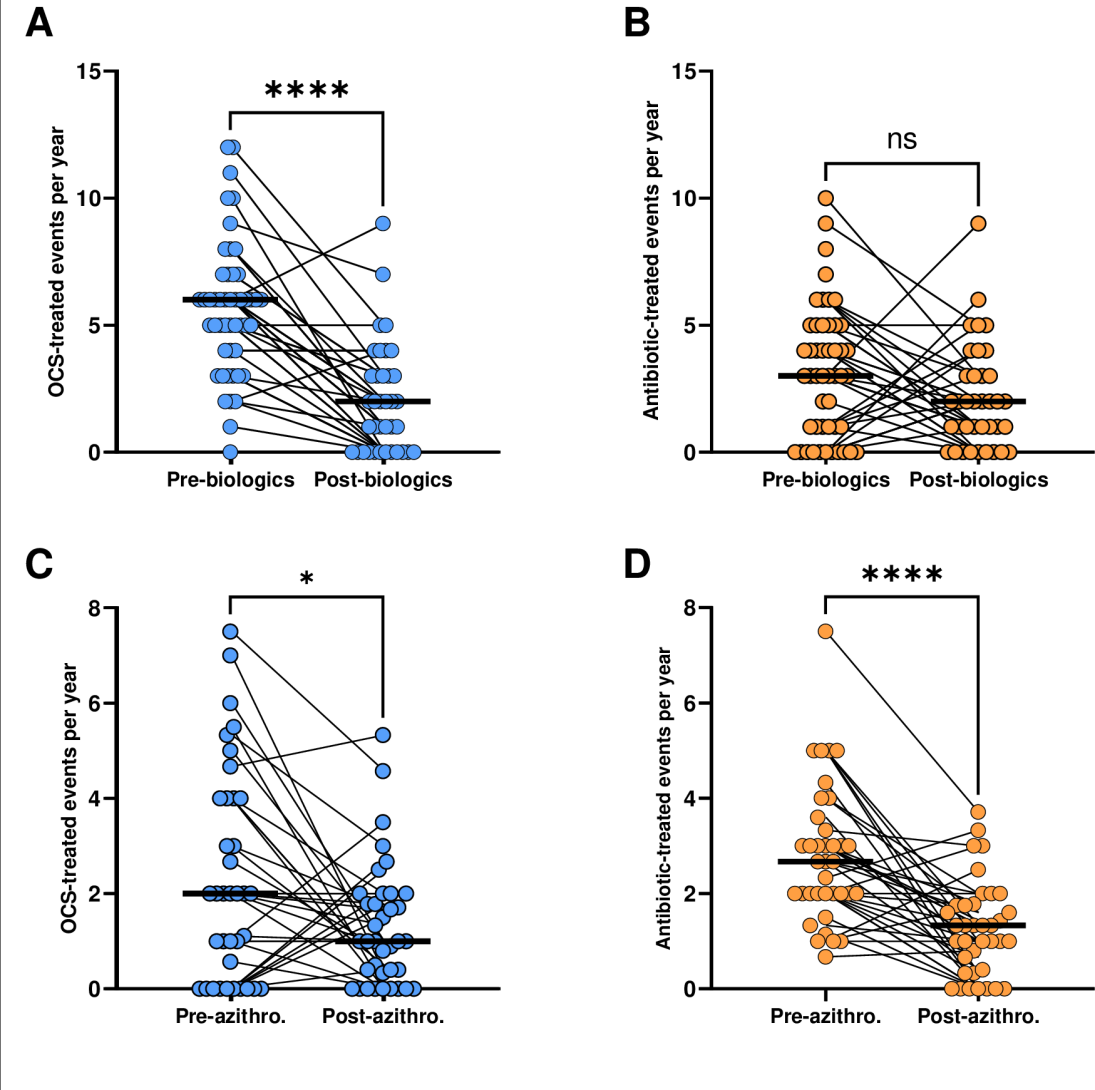
Change in median annual rates of oral corticosteroids (OCS) and antibiotic-requiring events before and after biologics and before and after azithromycin. **A** and **B** Biologics reduced the median rate of OCS-treated events from 6 to 2 median difference (95% CI) of 4.0 (2.0 to 5.0) events/year (p<0.001)), but not of antibiotic-treated events (change from 3 to 2, median difference 2.0 (0.0 to 2.5) events/year (p=0.09)). **C** and **D** Azithromycin reduced the median rates of OCS-treated events from 2 to 1 (median difference of 0.3 (0.0 to 1.9) events/year (p<0.05)) and antibiotic treated-events from 2 to 1 (median difference 1.6 (1.0 to 2.0) events/year (p<0.001)).

For the group that was treated with azithromycin, OCS-treated events saw a mean reduction of 3.4 (95% CI 2.4 to 4.4 events/year) following biologics (figure 1A, p<0.001). Antibiotic-treated events showed a mean reduction of 0.9 (95% CI −0.2 to 2.0 events/year) (figure 1B, p=0.09). By comparison, subjects in the comparator group had significant reductions in both OCS and antibiotics-treated events following biologics, with a mean difference of respectively 3.9 (95% CI 3.4 to 4.7 events/year) for OCS-treated and 1.9 (95% CI 1.3 to 2.6 events/year) for antibiotics-treated events (p<0.001 for both).

Paired data at least 12 months after starting azithromycin were available for 37 patients. Treatment with azithromycin produced a further significant reduction in both OCS and antibiotic-treated events with a mean reduction of 1.0 (95 % CI 0.3 to 1.8 events/year) (figure 1C, p=0.02) and 1.5 (95% CI 1.0 to 2.1 events/year) respectively (figure 1D, p<0.001).

Sensitivity analyses looking at differences in exacerbations before and after biologics and before and after azithromycin were conducted using only the individuals with at least 12 months between the start of biologics and the start of azithromycin (n=23) and for individuals who never switched biologics after starting azithromycin (n=30), both showing nearly identical treatment effect to the full group of subjects who had started azithromycin.

Azithromycin had a significant impact on symptoms (ACQ-5 score), with mean (SD) values improving from 2.91±1.43 to 1.41±1.16 (mean difference −0.95; 95% CI 0.1 to 1.8, p=0.04). Treatment did not significantly impact FEV_1_ (mean difference 0.05; 95% CI −0.13 to 0.23, p=0.547).

There was no obvious relationship between baseline characteristics and treatment response to azithromycin. There was a trend for increased effect in patients with a low (<20 ppb) fractional exhaled nitric oxide (FeNO) prebiologics (p=0.107) and for those with a positive pathogen on sputum cultures (p=0.100). Multivariate analysis including age, sex, type of biologic at start, smoking history, presence of chronic bronchitis and/or purulent exacerbations, presence of bronchiectasis, use of OCS, baseline FeNO and baseline blood eosinophil count was conducted. No variable predicted response to biologics or to azithromycin.

Safety data are presented in table 2. Gastrointestinal side effects were common, but liver function abnormalities and hearing issues were rare and self-limited, with the caveat that data was not systematically collected in patient records. No cases of QT interval prolongation were identified.

**Table 2:** Side effects after starting azithromycin

Characteristic	Azithromycin started (n=50)
Gastrointestinal side effects	
Minor/self-limited	13 (26%)
Led to treatment discontinuation	2 (4%)
ECG on azithromycin	
Normal	14 (28%)
Incomplete RBBB	1 (2%)
No ECG available	35 (70%)
QTc on azithromycin	
Median, IQR	424 (407–448)
Maximal	454
Liver function tests	
Normal	15 (30%)
Elevated	4 (8%), 3 with other causes*
Not available	31 (62%)

* 1 present before starting azithromycin, 1 CMV/EBV co-infection and 1 concomitant methotrexate at time of elevation.

QTCcorrected QT intervalRBBBright bundle branch block

## Discussion

In our cohort of patients with severe asthma and residual exacerbations despite biologics, additional azithromycin treatment resulted in a significant reduction in both steroid and antibiotic-requiring events, as well as significant improvements in ACQ-5 but not lung function. These results are consistent with other studies of macrolides in less severe asthma^2 3^ and suggest these findings can be generalised to include a biologic-treated severe asthma population.

Patients in our cohort who started azithromycin were older and had more exacerbations prior to biologics and did not show significant reduction in antibiotic-treated events following biologics unlike the rest of our severe asthma population. The demographic characteristics that differed most from the comparator cohort were daily sputum production (chronic bronchitis) and/or frequent purulent exacerbations, symptoms that are suggestive of persistent airways infection. We were interested in whether low baseline FeNO and/or positive sputum cultures were associated with a greater azithromycin effect, as FeNO has been shown to be inversely proportional to rates of Proteobacteria in the airway, including *Haemophilus influenzae*.^6 7^ There was a trend for a greater azithromycin effect in this group, but airway microbiology was not assessed systematically in our cohort, and we acknowledge that this may have compromised our ability to show an effect. Our audit has other important limitations, including being from a single centre, small sample size, non-randomised nature of azithromycin treatment and retrospective assessment of outcomes, limitations that are inherent to the study design. Larger randomised prospective trials incorporating more systematic assessment of both airway microbiology and type 2 biomarkers, preferably at baseline and at exacerbation, are needed to assess the response to azithromycin and associated factors in this population. Nonetheless, our findings provide a strong basis for these studies.

In summary, our findings suggest that azithromycin should be considered in patients with severe asthma treated with biologics with residual exacerbations and symptoms of chronic bronchitis and/or purulent exacerbations. Further research is required to confirm this and validate predictors of response to treatment.
